# Metabolomic analysis of human plasma sample after exposed to high altitude and return to sea level

**DOI:** 10.1371/journal.pone.0282301

**Published:** 2023-03-29

**Authors:** Jiayue Gao, Ming Zhao, Xiang Cheng, Xiangpei Yue, Fangbin Hao, Hui Wang, Lian Duan, Cong Han, Lingling Zhu

**Affiliations:** 1 Beijing Institute of Basic Medical Sciences, Beijing, China; 2 The Fifth Medical Centre, Chinese PLA General Hospital, Beijing, China; 3 Co-Innovation Center of Neuroregeneration, Nantong University, Nantong, China; Xiangtan University, CHINA

## Abstract

When ascending to high altitude, it is a rigorous challenge to people who living in the low altitude area to acclimatize to hypoxic environment. Hypoxia exposure can cause dramatic disturbances of metabolism. This longitudinal cohort study was conducted to delineate the plasma metabolomics profile following exposure to altitude environments and explore potential metabolic changes after return to low altitude area. 25 healthy volunteers living in the low altitude area (Nor; 40m) were transported to high altitude (HA; 3,650m) for a 7-day sojourn before transported back to the low altitude area (HAP; 40m). Plasma samples were collected on the day before ascending to HA, the third day on HA(day 3) and the fourteenth day after returning to low altitude(14 day) and analyzed using UHPLC-MS/MS tools and then the data were subjected to multivariate statistical analyses. There were 737 metabolites were obtained in plasma samples with 133 significantly changed metabolites. We screened 13 differential metabolites that were significantly changed under hypoxia exposure; enriched metabolic pathways under hypoxia exposure including tryptophan metabolism, purine metabolism, regulation of lipolysis in adipocytes; We verified and relatively quantified eight targeted candidate metabolites including adenosine, guanosine, inosine, xanthurenic acid, 5-oxo-ETE, raffinose, indole-3-acetic acid and biotin for the Nor and HA group. Most of the metabolites recovered when returning to the low altitude area, however, there were still 6 metabolites that were affected by hypoxia exposure. It is apparent that high-altitude exposure alters the metabolic characteristics and two weeks after returning to the low altitude area a small portion of metabolites was still affected by high-altitude exposure, which indicated that high-altitude exposure had a long-term impact on metabolism. This present longitudinal cohort study demonstrated that metabolomics can be a useful tool to monitor metabolic changes exposed to high altitude, providing new insight in the attendant health problem that occur in response to high altitude.

## Introduction

The high altitude plateau environment is an ecological environment characterized by hypobaric oxygen, low temperature and high ultraviolent radiation et al, in which the hypobaric oxygen is the major feature. High altitude forces the body to reduce the arterial oxygen partial pressure and oxygen saturation, eventually leading to tissue hypoxia, and then cause metabolic change. When humans enter the high altitude plateau environment from the low altitude area, physiological responses including ventilation function, cardiac function, hematology and oxygen consumption to help the body acclimation to hypoxia condition [1–4]. In addition, hypoxia induce the metabolic response to help match ATP synthesis and demand in the face of decreased oxidative capacity and increased oxidative stress [5].

Metabolomics has been proven as a powerful tool relying on analysis platforms to assess metabolic responses stimulated by external disturbance and environmental stimulation, further reflects the alteration of physiological and biochemical processes in the body [6]. For lowlanders who are access to high altitude, there are also remarkable metabolic responses to progressive environmental hypoxia. O’Brien et al revealed decreasing isoleucine with ascent alongside increasing lactate and decreasing glucose, which may towards increased glycolytic rate [7]. As reported previously, there is genetically-derived metabolic adaptation in Himalayan Sherpas, that allow their tissues to use oxygen more efficiently and conserve muscle energy at high altitude [8].

Metabolomics has been widely used to identify biomarkers in the field of high altitude medicine [9]. Based on clinical samples, it is found that hypoxia at high altitude has a dramatic and comprehensive effect on metabolites, and the changes in metabolites in the body vary under different hypoxia environment. Human plasma time-dependent metabolic profile revealed that amino acid metabolism, glycolysis and purine metabolism altered, and the time course of these changes is different over a two-week period exposed to simulated altitude exposure at 3000m [10]. When exposed to hypoxia at an altitude of 5300m, a plasma metabolomic study using 60 subjects showed that several key metabolic pathways were perturbed, including inflammatory response-related metabolism, energy metabolism, bile acid metabolism and heme metabolism [11]. Within the AltitudeOmics project, metabolomics results of red blood cells indicated that the changes of metabolic pathways after exposure to high altitude (5260m) were correlated to physiological and athletic performance parameters [12]. The nontargeted metabolomic analyses of muscle biopsies suggested an enhancement of muscle bioenergetics in physiological hypoxia, including glycolytic intermediates, amino acids and fatty acids [5]. Most people go to high altitude often accompanied by the occurrence of acute mountain sickness (AMS). For the plasma samples from individuals with and without AMS, Zhu *et al* found that hypoxanthine, cysteinylglycine, D-arabitol, L-allothreonine, 2-ketobutyric acid and succinate semialdehyde in the plasma from the AMS patients were increased significantly [13]. Changes in energy metabolism detected by urine metabolomics may contribute to the susceptibility to AMS at high altitude (4300m) [14].

Generally, most of the existing studies are based on cross-sectional studies, and there are few longitudinal cohort studies. Also, there are limited reports on the metabolic changes after returning from the high altitude plateau to the low altitude area. Physiological indicators are recovered easily from exposure to hypobaric hypoxia after returning to the low altitude environment such as heart rate, ventilation, O_2_ uptake, and arterial oxygen saturation [12, 15, 16], however, it is not clear about the recovery of the metabolism changing. Recent advances in Metabolomic techniques make it possible to uncover plasma metabolic profiles in individuals, which will provide a better insight into the hypoxia adaptation as well as recovery from hypoxia stimulation.

In the present study, we performed a non-targeted metabolomics analysis based on ultra-high performance liquid chromatography mass spectrometry (UPLC-MS/MS) to investigate the metabolic alterations when exposed to hypoxia at high altitude as well as two weeks later after the exposure.

## Materials and methods

### Ethical approval

This study was approved by the Human Research Ethics Committee at Beijing Institute of Basic Medical Sciences. Written informed consent was obtained from all participants before participation in the study.

### Participants

Twenty five volunteers (fifteen men and ten women) who had not been to the high altitude plateau in the past six months were recruited in this study (mean ± SD age 29.7 ± 5.8 years; body mass 63.6 ± 9.7 kg). The basic characteristics of the volunteers were summarized in Table 1. Participants were instructed to maintain a regular diet during the study, but to refrain from caffeine and alcohol on the day of blood sampling. All participants self-reported compliance with these requirements. Blood samples were collected on the day before Lhasa (3650m), the third day on Lhasa and the fourteenth day after returning to low altitude [17]. We have performed statistics and found that there are no gender differences. In HA group, oxygen saturation decreased and heart rate increased significantly without gender differences, which was consistent with that reported in literatures [18, 19].

**Table 1:** Basic characteristics of volunteers.

	Nor group		HA group		HAP group	
	Males	Females	Males	Females	Males	Females
	(n = 15)	(n = 10)	(n = 15)	(n = 10)	(n = 15)	(n = 10)
Site of sampling	Left ventricle	Left ventricle	Left ventricle	Left ventricle	Left ventricle	Left ventricle
Oxygen saturation (%)	97.9	97.7	82.7	82.1	98.1	97.9
	(96–100)	(96–99)	(77–88)*	(77–87)*	(97–99)	(97–99)
Heart rate (bpm)	84.8	86.2	102.1	104.2	85.4	86.4
	(63–99)	(76–100)	(89–115)*	(89–125)*	(71–94)	(79–94)
Red blood cell (×10^12^/L)	5.17	4.93	5.29	5.09	5.19	4.97
	(4.46–6.17)	(4.48–5.32)	(4.62–6.71)	(4.60–5.65)	(4.61–6.47)	(4.56–5.38)
Hemoglobin (g/L)	155.5	146.3.8	160.0	153.1	156.0	148.9
	(130–171)	(127–166)	(136–178)	(143–163)	(137–170)	(132–173)

* Represents p<0.05 values vs Nor group.

### Biological specimens and chemical reagents

Methanol and formic acid (FA) were chromatography grade and purchased from Thermo Fisher (USA). Ultrapure water was purchased from Merck (Germany).

### Sample collection and preparation

Plasma samples were collected from 25 healthy volunteers. Morning fasting blood was drawn into 5 mL EDTA tubes (SST; containing clot activator and plasma separator gel) (BD Bioscience, Franklin Lakes, NJ, USA) to prepare plasma. Samples were centrifuged at 3000 rpm for 10 min to obtain the plasma. 400 μL methanol mixed with 100 μL of plasma using vortex for 30s to precipitate protein. Supernatant were obtained by centrifuging at 15,000 rpm for 10 min at 4°C. 255 μL analytical grade water was added into the supernatant to make methanol concentration to 53%, then centrifuged (15,000 rpm 4°C, 10 min) prior to LC-MS analysis.

Quality control samples were generated by taking equal volume samples from each test sample and mix them. QC samples were used for the assessment of instrument stability and data quality throughout the study.

### UHPLC-MS/MS analysis

LC used Vanquish UHPLC system (Thermo Fisher, Germany) and a Hypesil Gold column (100×2.1 mm, 1.9μm) (Thermo Fisher, Germany) with a 17-min linear gradient at a flow rate of 0.2 mL/min. The eluents for the positive polarity mode were eluent A (0.1% FA in Water) and eluent B (Methanol).The eluents for the negative polarity mode were eluent A (5 mM ammonium acetate, pH 9.0) and eluent B (Methanol).The solvent gradient was set as follows: 2% B, 1.5 min; 2–100% B, 12.0 min; 100% B, 14.0 min; 100–2% B, 14.1 min; 2% B, 17 min. Injected sample volumn was 8 μL and the column oven was maintained at 40°C. LC was coupled with an Orbitrap Q Exactive^TM^ HF-X mass spectrometer (Thermo Fisher, Germany). Q Exactive^TM^ HF-X mass spectrometer was operated in positive polarity mode with spray voltage of 3.2 kV, capillary temperature of 320°C, sheath gas flow rate of 40 arb and aux gas flow rate of 10 arb. Full scan mass spectrometry data were acquired in a mass range of m/z 70–1050.

### Data processing and statistical analyses

The raw data files generated by UHPLC-MS/MS were processed using the Compound Discoverer 3.1 (CD3.1, Thermo Fisher) to perform peak alignment, peak picking, and quantitation for each metabolite. Peaks with a signal-to-noise ratio (S/N) greater than 50 were considered and normalized to the intensity of the total spectral intensity. The normalized data was used to predict the molecular formula based on additive ions, molecular ion peaks and fragment ions. Metabolite identifications were established by matching against online spectral libraries (mzCloud, mzVault and MassList) to obtain the accurate qualitative and relative quantitative results.

These metabolites were annotated using the KEGG database (https://www.genome.jp/kegg/pathway.html), HMDB database(https://hmdb.ca/metabolites) and LIPID Maps database (http://www.lipidmaps.org/). Principal components analysis (PCA) and Partial least squares discriminant analysis (PLS-DA) were performed at SIMCA 13.0 software (Umetrics) treated with univariate scaling (UV). We applied univariate analysis (t-test) to calculate the statistical significance (*P*-value). The metabolites with VIP > 1 (VIP: variable importance in projection) and *P*-value< 0.05 and FC≥ 2 or FC≤ 0.5 (FC: fold change)were considered to be differential metabolites. Volcano plots were used to filter metabolites of interest which based on log2 (FC) and -log10 (*P*-value) of metabolites.

## Results

### 1. Plasma metabolic profiling assayed by UHPLC-MS/MS

**Fig 1:**
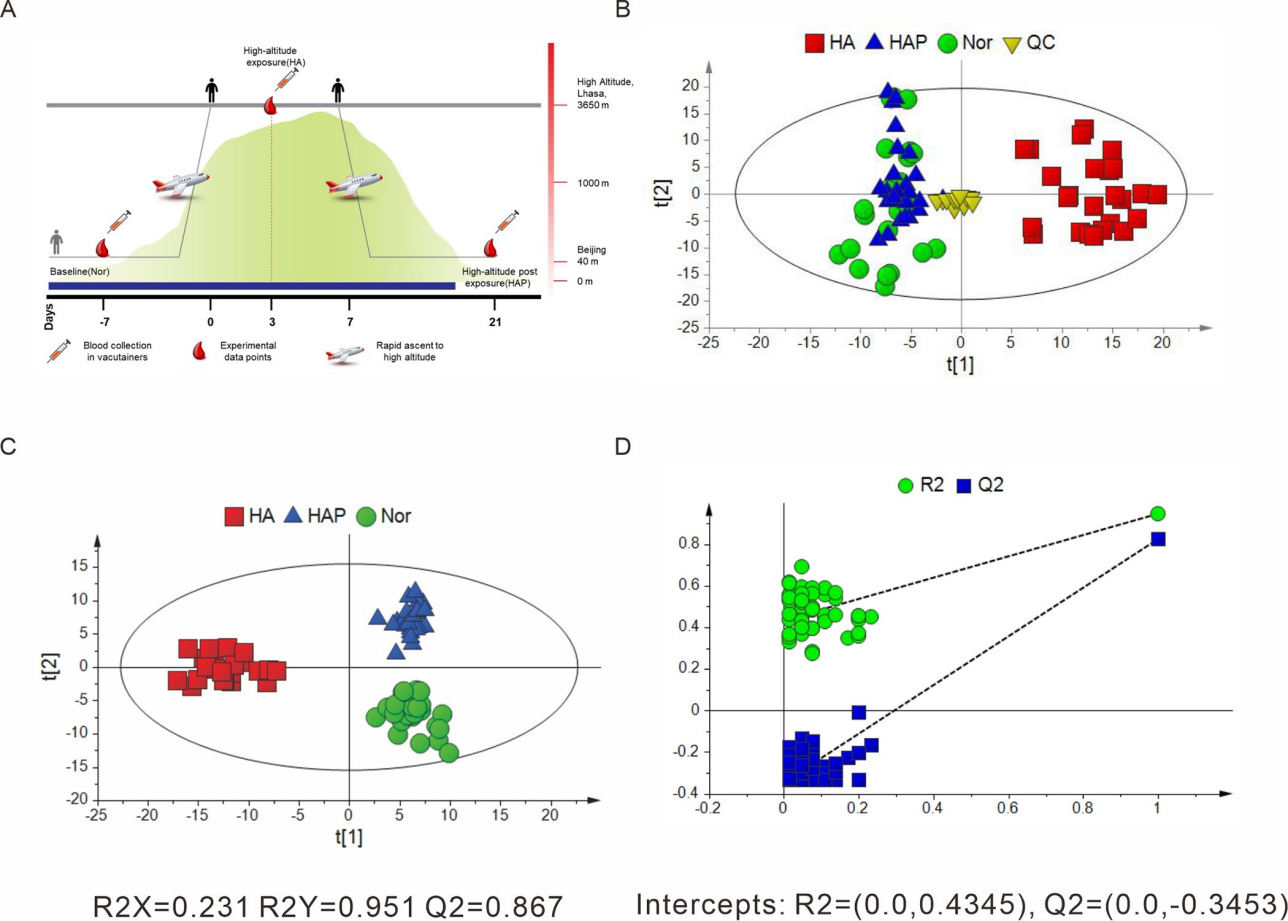
The metabolic profiles of plasma samples. Schematic diagram of sample taking (A). PCA (B) and PLS-DA (C) score plots for Nor, HA and HAP based on metabolomics data and plot of permutation tests (n = 50) of PLS-DA model for plasma profiles detected with LC-MS.

In this study, a total of 25 healthy individuals with ages from 20 to 45 were recruited. The study comprised three phases: baseline phase (before going to the highland, Nor), high altitude phase (going to the high altitude plateau, HA) and high-altitude-post phase (going back to the low altitude area, HAP). Plasma sample was collected 7 days before going to the high altitude plateau, 3 days after going to the high altitude plateau and 14 days after returning to the low altitude area (Fig 1A). Total ion chromatograms (TIC) of each group were shown in S1 Fig in S1 File. The data acquired was used to establish principal components analysis (PCA) model, an unsupervised pattern recognition, after peak alignment and normalization. The PCA score plot (Fig 1B) showed that QC samples clustered together tightly, indicating great QC repeatability and analysis system stability, and the metabolic profiles of HA and Nor group were apparently separated. In contrast, the profiles of HAP cannot be distinguished from HA group, indicating that the characteristics of HA resembled that of HAP. The PCA figure provided an overview of all the groups, however the variables responsible for differences in each cluster were still unclear. Supervised pattern recognition like partial least squares-discriminant analysis (PLS-DA) model was employed in finding differentiated metabolites. PLS-DA model (Fig 1C) explained 95.1% original data with powerful prediction ability (Q2 = 0.867). The permutation plot with a negative intercept of Q2 regression line (Fig 1D) indicated that the original PLS-DA model was efficient and reliable with a low risk of over fitting. The PLS-DA showed an overall trend of shifting, which indicates alteration in metabolic process. PLS-DA reveals more prominent trend of change. The points for HA (red square) are separated from those for Nor (green circle) and HAP (blue triangle), which shows that the metabolic profiling of Nor and HAP were more similar, with understandable reason that the samples were all collected in the low altitude environment. However, the metabolic profiling of Nor and HAP existed differences for that Nor and HAP were distinguished on the second principal component.

### 2. Screening of differential metabolites upon exposure to 3-day high altitude

**Fig 2:**
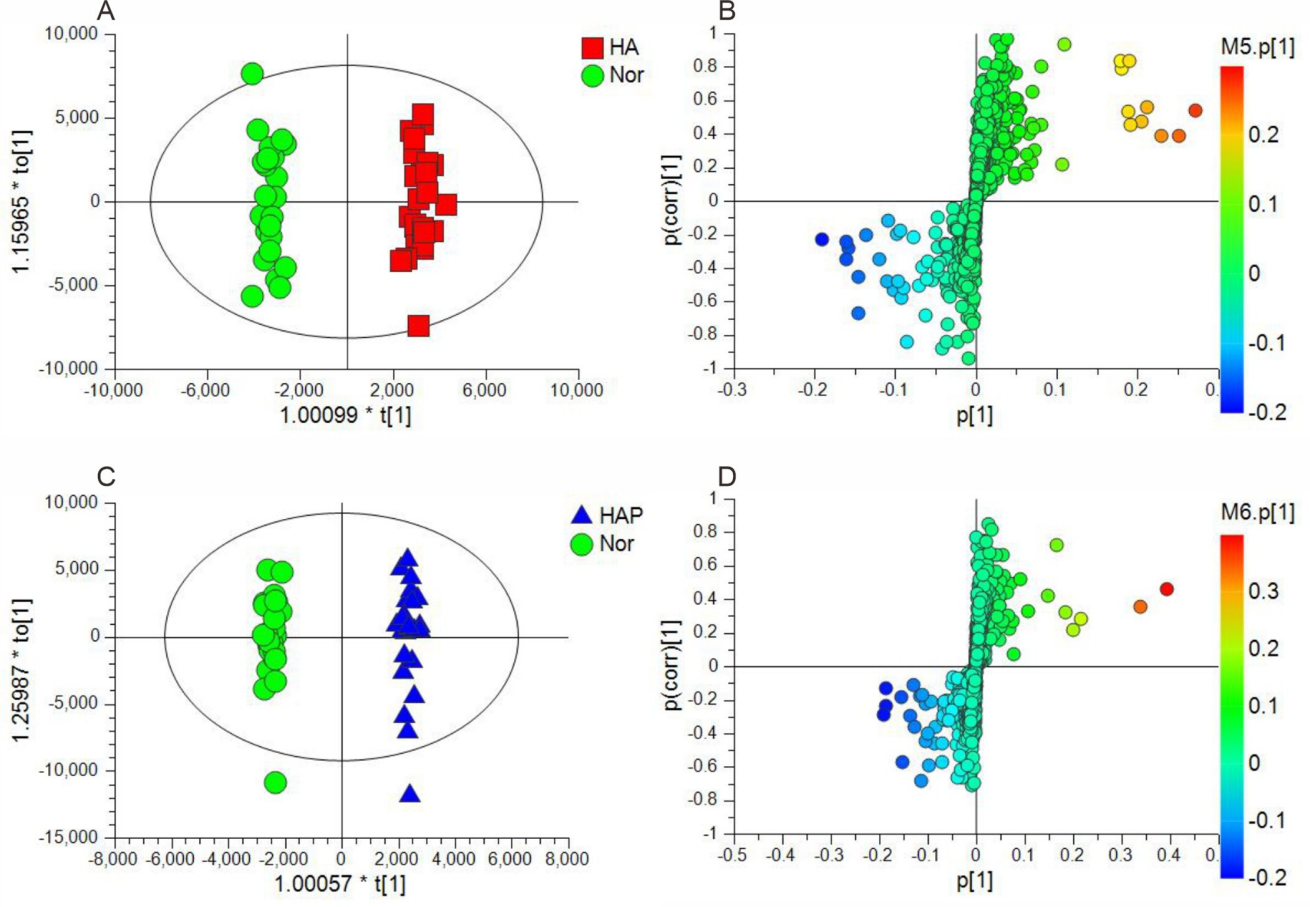
OPLS-DA analysis of the metabolic samples. Panels A and C represents the comparison of the HA and HAP group with the Nor group respectively. Panels B and D are S-Plot diagrams obtained by comparing HA and HAP respectively.

There were 737 metabolites were obtained in plasma samples. In order to compare the differences of HA and Nor groups, We re-modeled the data to investigate the effect of high altitude and high-altitude-post (HAP) on plasma metabolism in human (Fig 2). OPLS-DA analysis was performed on the pairwise metabolite data with par scaling, consisting of Nor, HA and HAP. We used the S-plot method for each group to screen out differential metabolites. The S-plot is a scatter plot that combines the covariance and correlation loading profiles resulting from a projection-based model, which visualizes the variable influence of model [20]. The abscissa of the S-plot represents the load of each substance on the first principal component, and the ordinate represents the correlation coefficient between each substance and the first principal component.

**Fig 3:**
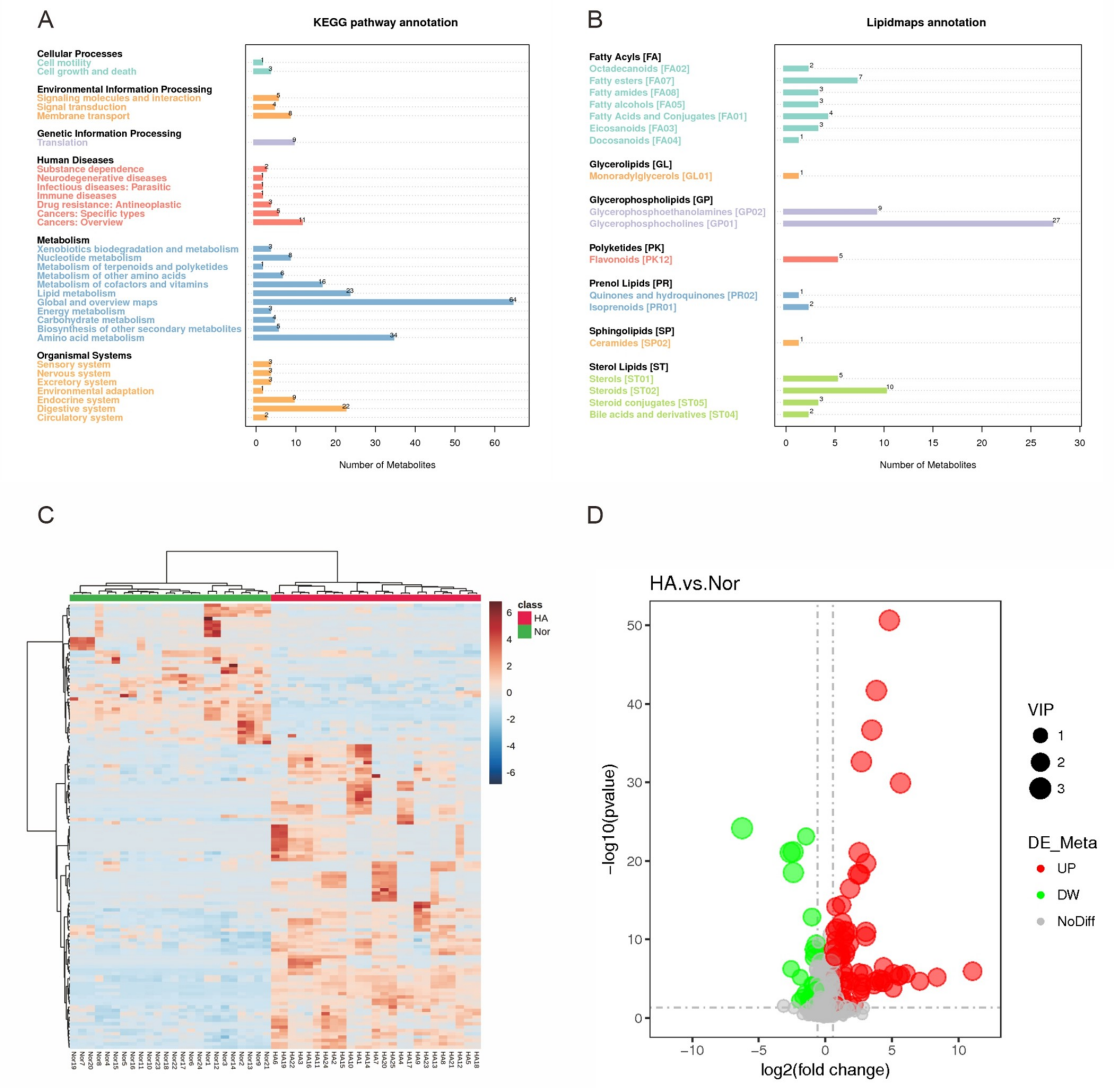
Annotation and screening of metabolites. Annotation of metabolite pathway results based on KEGG (A) and Lipidmaps (B). Heat map of the differential metabolites between Nor and HA groups (C). The volcano plot of the metabolites, red points represent up-regulated metabolites and green point represented down-regulated metabolites (D).

We screened out 133 significantly changed metabolites with a criteria of VIP >1.0 and *P* < 0.05. The differentiated metabolites were classified according to the pathway types in KEGG and Lipidmaps as shown in Fig 3A and 3B, which were used to create heatmap hierarchical clustering (Fig 3C). The heat maps showed that Nor & HA existed differences in metabolic profiling. Compared HA with Nor group, there are 91 up-regulated metabolites and 42 metabolites down-regulated in volcano plot (Fig 3D). We further screened differential metabolites with more stringent criteria of VIP >1.0, *P* < 0.01, fold change (FC) >1.5 and AUC>0.7 (ROC) (Table 2). The metabolites that changed significantly mainly included lipid and lipid derivatives, adenosine, inosine and melatonin.

**Table 2:** The differential metabolites between Nor and HA.

No.	Metabolite	Retention Time (min)	VIP	FC	*P* value	ROC	Up/Down
**1**	Decanoylcarnitine	11.72	5.28	1.71	6.88E-06	0.85	up
**2**	Monoolein	15.06	5.23	3.61	3.42E-17	1	up
**3**	Indole-3-acetic acid	9.57	3.90	0.5	5.96E-05	0.85	down
**4**	2-Mercaptobenzothiazole	11.27	2.98	8.33	2.11E-20	1	up
**5**	PC (18:5e/2:0)	14.22	2.76	0.54	6.81E-05	0.83	down
**6**	Adenosine	2.64	2.34	0.01	7.06E-25	1	down
**7**	Melatonin	9.81	1.95	333.48	6.78E-06	0.81	up
**8**	PC (15:0/15:0)	16.17	1.65	0.58	6.13E-09	0.94	down
**9**	PE (16:1e/22:5)	16.29	1.58	0.62	2.29E-04	0.83	down
**10**	Tetrahydrocorticosterone	12.53	1.50	1.66	1.47E-05	0.82	up
**11**	Inosine	3.09	1.16	0.19	3.09E-19	1	down
**12**	5-OxoETE	13.87	1.10	1.53	1.22E-03	0.77	up
**13**	2-Arachidonoyl glycerol	14.97	1.07	1.59	1.20E-04	0.84	up

### 3. Metabolic pathway analysis

**Fig 4:**
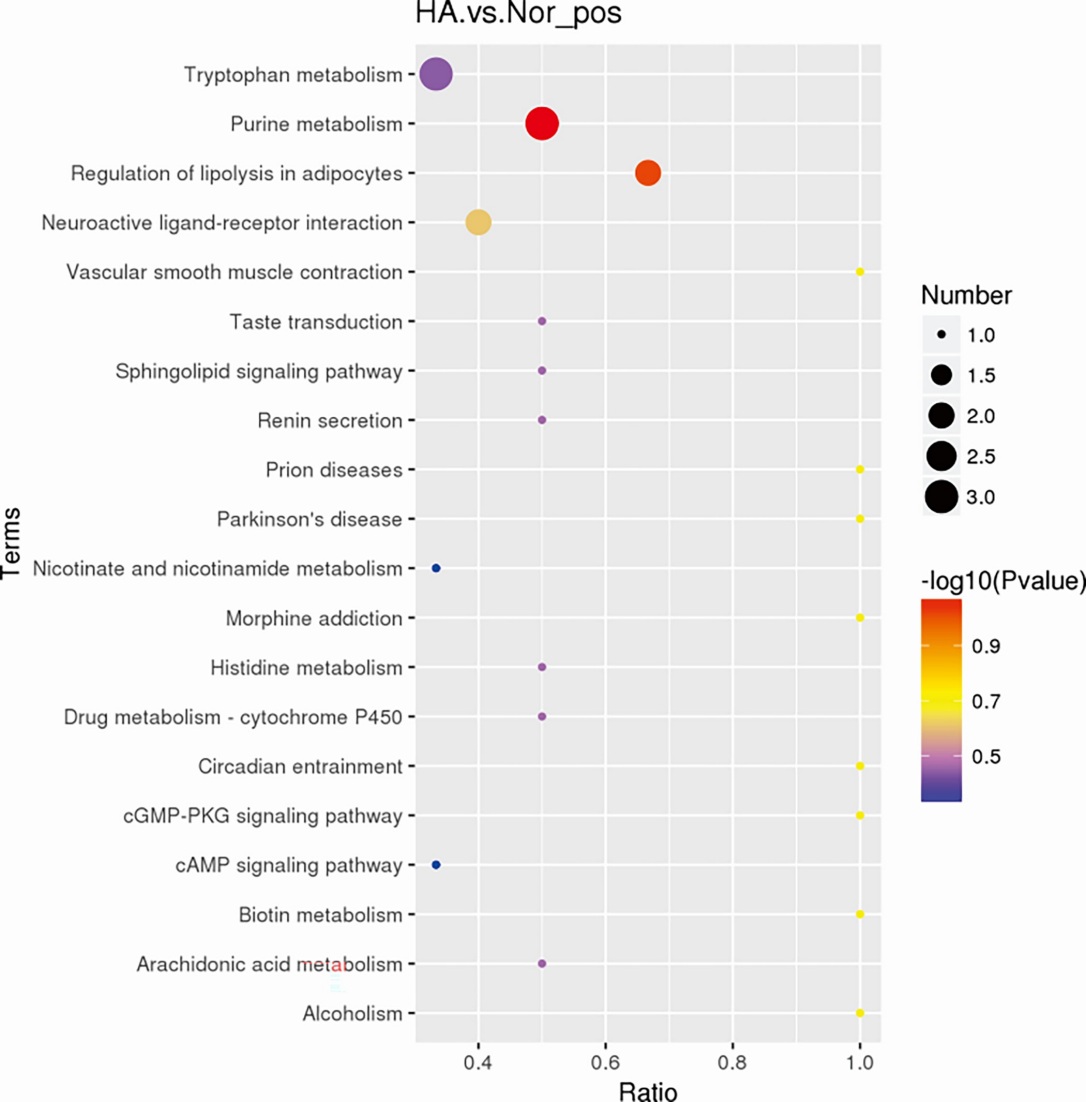
Enrichment analysis of significant metabolic pathways related to the HA group.

Functional enrichment analysis was performed to determine the most relevant pathways involved in HA group as shown in Fig 4 and Table 3. According to the enrichment of pathways of the altered metabolites, we screened the following dysregulated metabolic pathways-tryptophan metabolism, purine metabolism, regulation of lipolysis in adipocytes, neuroactive ligand-receptor interaction, etc. Most of the pathways were associated with nucleotides, including xanthurenic acid, guanosine, adenosine and inosine indicating the altered amino acids in these pathways have high potential to be HA biomarker candidates. Besides, indole-3-acetic acid, a breakdown product of tryptophan metabolism, 5-oxo-ETE, a kind of arachidonic acid, raffinose were enriched in the metabolism pathways in the HA group.

**Table 3:** Affected metabolic pathways in HA.

MapTitle	*P* value	Metabolites
Purine metabolism	0.084	Guanosine, Adenosine, Inosine
Regulation of lipolysis in adipocytes	0.095	Adenosine, Corticosterone
Biotin metabolism	0.194	Biotin
cGMP-PKG signaling pathway	0.194	Adenosine
Vascular smooth muscle contraction	0.194	Adenosine
Circadian entrainment	0.194	Melatonin
Parkinson’s disease	0.194	Adenosine
Prion diseases	0.194	Corticosterone
Morphine addiction	0.194	Adenosine
Alcoholism	0.194	Adenosine
Neuroactive ligand-receptor interaction	0.247	Adenosine, Melatonin
Histidine metabolism	0.351	1-Methylhistidine
Arachidonic acid metabolism	0.351	5-OxoETE
Drug metabolism—cytochrome P450	0.351	2,6-Xylidine
Sphingolipid signaling pathway	0.351	Adenosine
Taste transduction	0.351	D-Phenylalanine
Renin secretion	0.351	Adenosine
Tryptophan metabolism	0.369	Xanthurenic acid, Indole-3-acetic acid, Melatonin

### 4. Metabolites in HAP partially recovered

**Fig 5:**
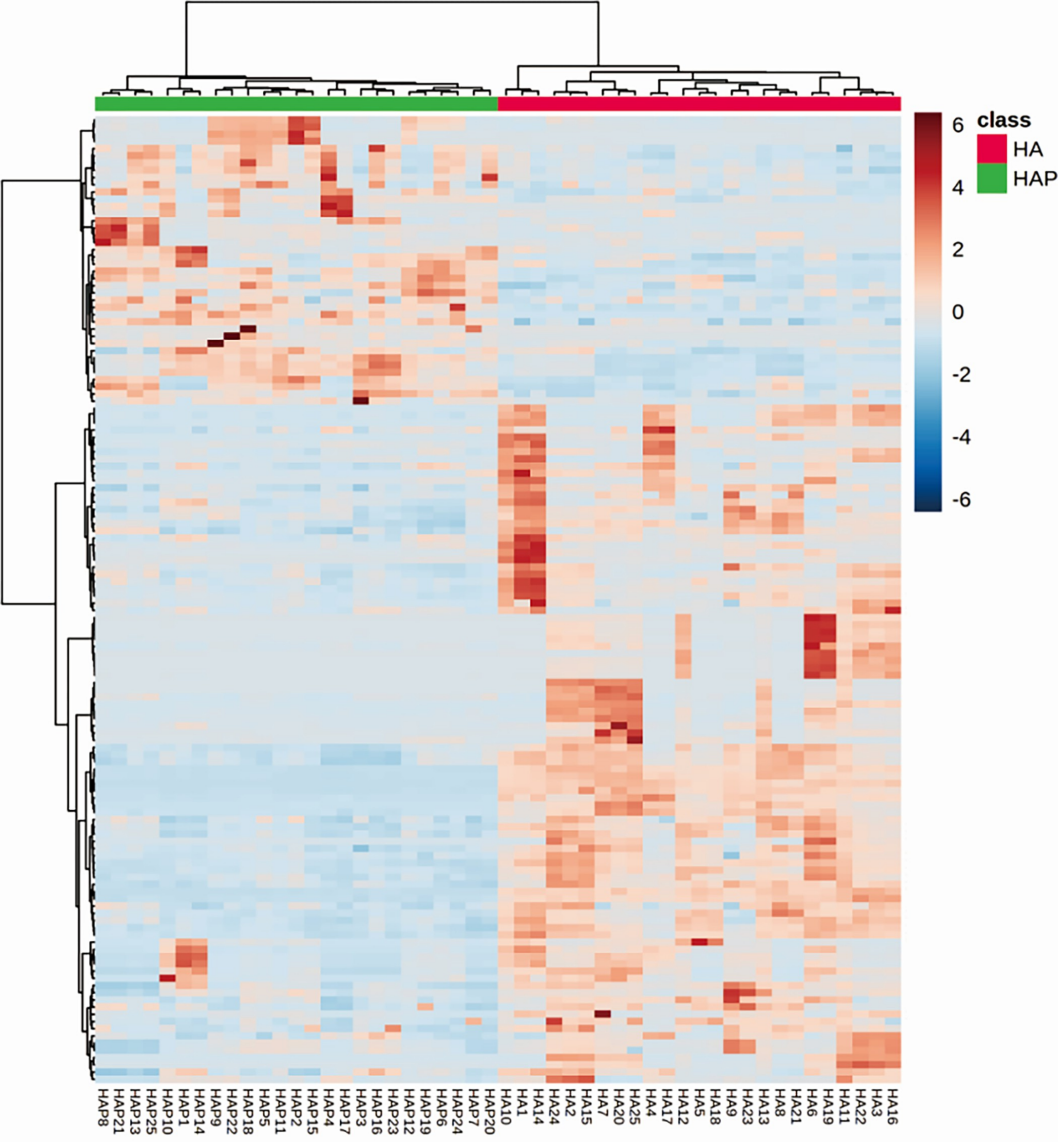
Heat map of the differential metabolites between HA and HAP groups.

After returning to the low altitude environment from the high altitude plateau, the partial pressure of oxygen changed again and we assumed that the metabolic situation would change again and the metabolites would partially recover. In this study, we analyzed the data of high-altitude-post. Heatmap hierarchical clustering analysis of 135 different metabolites between HA and HAP was carried out and the results showed that there were significant differences in metabolites between the two groups (Fig 5). We further screened differential metabolites between HA and HAP with rigorous criteria as in HA/HAP (Table 4). As the data shown that adenosine level was decreased in HA, which was consistent with the report [10] and then decreased increased partly in HAP. The content of monoolein, melatonin and phosphatidylcholines (PC) decreased firstly and then increased, showing the opposite trend. In HAP group, most of the acylcarnitine (Acar), which were related to the energy metabolism were down regulated. It showed that the level of metabolite recovered when returning to the low altitude environment, but it was still different from Nor group.

**Table 4:** The differential metabolites between HA and HAP.

No.	Metabolite	RT (min)	VIP	FC	*P* value	ROC	VIP	Up/Down
**1**	ACar 18:2	13.35	5.30	0.47	7.56E-20	1.00	2.90	down
**2**	ACar 18:1	13.57	5.29	0.52	1.07E-15	1.00	2.67	down
**3**	Decanoylcarnitine	11.72	4.84	0.59	1.31E-05	0.84	1.23	down
**4**	Monoolein	15.06	4.65	0.33	3.00E-14	0.99	2.18	down
**5**	2-Mercaptobenzothiazole	11.27	2.89	0.07	1.08E-33	1.00	3.01	down
**6**	Paraxanthine	6.81	2.57	0.52	2.65E-05	0.84	1.39	down
**7**	ACar 14:1	12.77	2.22	0.53	0.002798	0.87	1.29	down
**8**	Creatine	1.36	2.03	1.51	9.38E-06	0.84	1.37	up
**9**	Phytosphingosine	12.20	2.00	0.40	0.000177	0.77	1.11	down
**10**	Melatonin	9.81	1.83	0.00	4.78E-06	0.84	2.09	down
**11**	N-Phenylacetylglutamine	12.53	1.78	0.55	5.61E-07	0.87	1.70	down
**12**	ACar 16:1	13.21	1.61	0.50	6.14E-10	1.00	2.20	down
**13**	PC (16:0/17:0)	15.84	1.61	2.83	5.54E-06	0.83	1.91	up
**14**	Theophylline	6.96	1.42	0.53	2.98E-05	0.82	1.42	down
**15**	Adenosine	2.64	1.41	35.96	1.05E-18	1.00	2.46	up
**16**	ACar 13:0	12.69	1.36	2.03	3.07E-05	0.84	1.25	up
**17**	ACar 10:1	11.15	1.33	0.63	7.54E-06	0.86	1.20	down
**18**	Theobromine	6.13	1.33	0.43	0.000129	0.78	1.30	down
**19**	ACar 20:3	13.49	1.21	0.46	6.09E-16	1.00	2.61	down
**20**	ACar 18:3	13.14	1.19	0.45	9.80E-16	1.00	2.60	down
**21**	5-OxoETE	13.87	1.19	0.60	0.000216	0.80	1.41	down
**22**	ACar 20:4	13.34	1.15	0.48	0.00288	0.96	1.47	down
**23**	ACar 20:2	13.68	1.08	0.54	5.88E-17	1.00	2.57	down
**24**	ACar 26:1	14.52	1.07	0.48	7.07E-14	0.98	2.25	down
**25**	Perillartine	5.95	1.00	0.04	2.27E-52	1.00	2.86	down

## Discussion

In this study, the metabolic profiling of human plasma sample when exposed to low altitude area, 3 days of high altitude hypoxia and 14 days after returned to low altitude area were compared. Non-targeted metabolomics based on UHPLC-MS/MS was performed to investigate the metabolic changes, screen differentiated metabolites and metabolic pathways. Principal component analysis results indicated that the plasma metabolite profiles changed significantly as the subjects moved from low altitude area to high altitude and back to low altitude area, reflecting the subjects’ response to altitude changes.

**Fig 6:**
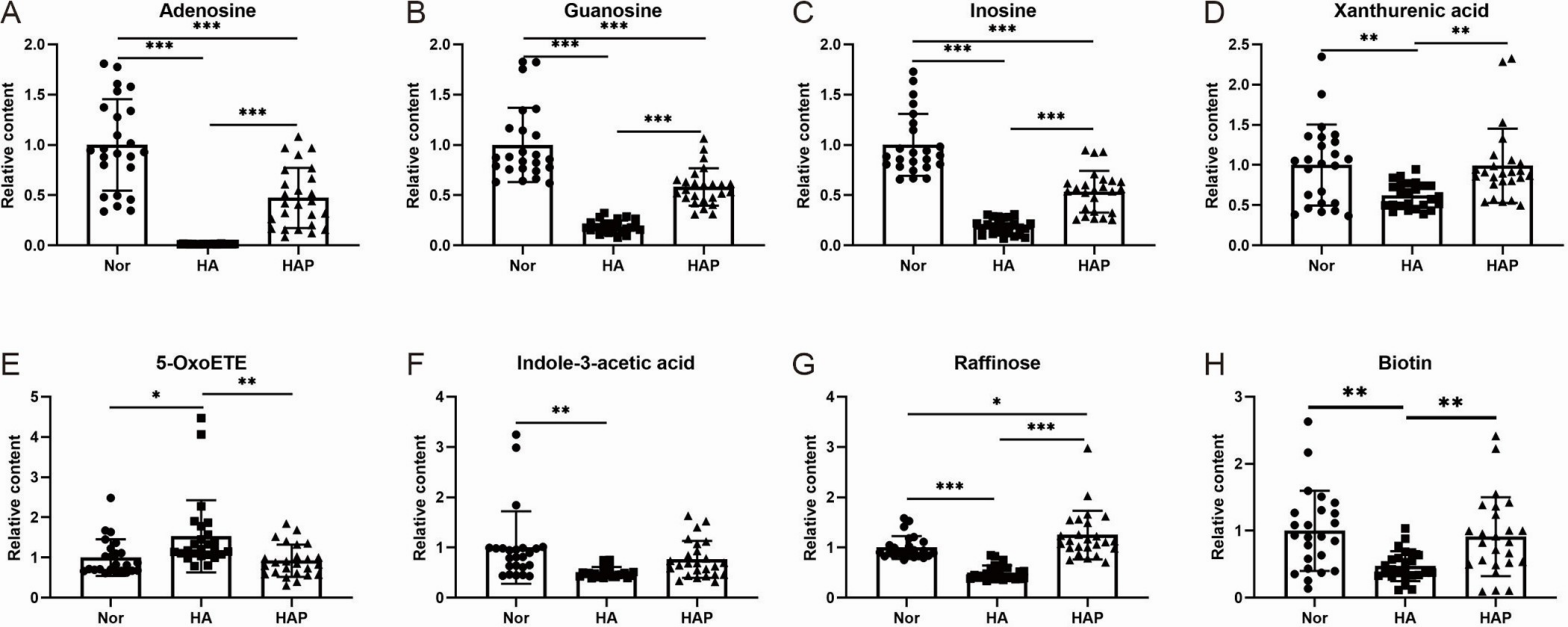
Plots representing the relative content of (A) Adenosine, (B) Guanosine, (C) Inosine, (D) Xanthurenic acid, (E) 5-OxoETE, (F) Indole-3-acetatic acid, (G) Raffinose, and (H) Biotin in three groups.

In the Mount Everest region, about 50% of trekkers who walk to altitude higher than 4000m develop AMS and 84% of people who fly directly to 3860 m are affected [21]. Several articles have reported the changes of metabolic level under hypoxia exposure at high altitude. Previous studies have showed that amino acid metabolism, glycolysis and purine metabolism altered, and the time course of these changes is different over a two-week period exposed to simulated altitude exposure at 3000m [10]. A plasma metabolomic study using 60 subjects showed that several key metabolic pathways were perturbed, including inflammatory response-related metabolism, energy metabolism, bile acid metabolism and heme metabolism at an altitude of 5300m [11]. In addition to plasma, population-based cross-sectional studies using red blood cells and muscle biopsies have also been reported. Metabolomics results of red blood cells indicated that the changes of metabolic pathways after exposure to high altitude (5260m) were correlated to physiological and athletic performance parameters. The researchers found that hypoxia promoted glycolysis, the pentose phosphate pathway and catabolism of purine and nitric oxide [12]. The skeletal muscle of lowlanders exposed to altitudes between 3000 and 5300 m, there are signals of metabolic modulation consistent with a suppression of oxygen demand, including down-regulation of mitochondrial electron transfer system complexes and tricarboxylic acid (TCA) cycle enzymes [22, 23]. There are still many differences between our study and previous studies. The ascending height of the subjects in our study is 3650m, which is lower than that in other studies. We performed plasma as metabolic samples for plasma can reflect the overall changes of the body under external environmental stimuli. In previous literatures, the samples used are different from ours, such as red blood cells, muscle tissue, urine, and so on, which may be responsible for the metabolic differences. Additionally, different sampling time may also lead to differences in screening metabolites. We took blood samples on the third day of high altitude exposure, and on the 14th day after we returned to the plain, with implications for the metabolic changes induced by acute hypoxia exposure and the long-term effects of metabolism after hypoxia exposure. In this cohort study, we found that purine metabolism was fluctuated with the partial pressure of oxygen. When ascending to high altitude, adenosine, guanosine and inosine in plasma were downregulated, while after returning to the low altitude area these metabolites were partial recovered (Fig 6). In addition, lipid metabolism also changed with PC and PE downregulated and decanoylcarnitine and 2-arachidonoyl glycerol upregulated when exposed to hypoxic environment and when back to the low altitude area the content of most acylcarnitines decreased compared with the high altitude plateau. In addition, acylcarnitines increased when exposed to hypoxia and then decreased after back to the low altitude area. That hypoxic exposure causes elevated carnitine levels was consist with previous findings [11, 24]. It is reported that carnitines could protect myocardial function [25] and brain function from hypoxia and oxidative stress [26, 27]. Administration of acetyl-L-carnitine attenuates neuronal damage, prevents apoptosis and improves energy status in hypoxic stress [27, 28]. We speculated that the carnitine system is essential for high altitude adaptation.

It is reported that intracellular adenosine triphosphate stores are rapidly depleted [29]. Consistently, we also found decreased levels of adenosine, guanosine, inosine and xanthurenic acid when exposed to high altitude hypoxia 3 days. Additionally, the relative content of adenosine, guanosine and inosine in HAP group were recovered to a certain extent when returned to the low altitude area 14 days, but did not recover completely, indicating that exposure of high altitude hypoxia exerted a long-term effect on the body metabolism (Fig 6). Nucleotides play important roles in biological processes and participating in cell signaling. In addition, adenosine belongs to a purinergic signaling molecules. Adenosine signaling could dampen inflammatory responses and subsequently protects tissues from unrestricted inflammatory [30, 31]. It is reported that adenosine deaminase maintains a high level of activity, and the production of adenosine increases significantly in the early stage of hypoxia. After adapting to hypoxia, the production of adenosine decreases, so that the metabolic rate of adenosine exceeds its production rate [32]. In addition, adenosine is constantly consumed due to its binding with receptor, which may be the reason for the decrease of adenosine in plasma.

Tryptophan is an essential aromatic amino acid in human body containing indole ring. It can be derived from dietary intake or endogenous protein degradation. Dietary tryptophan can be metabolized into indole-3-acetic acid (IAA) as by-products of the amino acid metabolism by gut microbiota through indole-3-acetamide pathway under the catalysis of tryptophan monooxygenase and indole-3-acetamide hydrolase [33]. Tryptophan metabolism through the kynurenine pathway is involved in the regulation of immunity, neuronal function and intestinal homeostasis. The level of IAA can be used to assess tryptophan metabolism pathway. It is reported that tryptophan metabolism was greatly reduced in synovial fibroblasts by hypoxia [34]. Xanthurenic acid is also involved in the tryptophan-catabolizing kynurenine pathway. Our results showed that IAA and xanthurenic acid were down-regulated in HA group, a low oxygen condition, which came back to initial level in HAP group (Fig 6). Tryptophan metabolism was affected by oxygen concentration. Hypoxia can exacerbate inflammation, and in severe cases it can lead to life threatening altitude sickness such as cerebral edema and pulmonary edema [35, 36]. We hypothesized that hypoxia may modulate inflammation through tryptophan metabolism pathway.

The potent eosinophil chemoattractant 5-oxo-6,8,11,14-eicosatetraenoic acid (5-oxo-ETE) is a 5-lipoxygenase product that acts mediated by the selective OXE receptor, which is present in many species [37, 38]. 5-Oxo-ETE is the major 5-HETE metabolite formed by neutrophils, in which the formation of NADPH was accompanied. In addition to neutrophils, 5-oxo-ETE is also produced by activation of the respiratory burst of eosinophils and monocytes. 5-oxo-ETE acts synergistically with many of lipid and peptide mediators which are involved in regulating the course of inflammation including other lipid mediators, chemokines, and cytokines [39]. Elevated 5-oxo-ETE (Fig 6), a potent eosinophil chemoattractant which can be catabolized in inflammatory cells and epithelial cells [40], may be related to high altitude adverse reactions.

Biotin is a co-enzyme for pyruvate carboxylase, propionyl-CoA carboxylase, 3-methylcrotonyl-CoA carboxylase and two isoforms of acetyl-CoA carboxylase, which involved in energy metabolism, fatty acid synthesis, and amino acid catabolism [41]. Due to the decrease of biotin content in HA group (Fig 6), the function of biotin as coenzyme may be weakened, which may lead to the change of metabolism.

This study demonstrates that metabolomics is a promising tool for discovery and understanding of novel biochemical responses to high altitude hypoxia exposure, providing new insight in the field of high-altitude medicine and the attendant health problems that occur in response to high altitude. The potential markers can be used to evaluate the physiological conditions and provide early warning for the early detection of high-altitude disease.

## Conclusion

This study observed the human plasma metabolites change as a result of hypoxia. 25 healthy volunteers were recruited in this study. From the metabolic profiles collected from each subject, high altitude hypoxia exposure affected the metabolism with many metabolites shifted. The levels of eight potential biomarkers demonstrated significant differences between HA group and Nor group, which recovered when returned to the low altitude area but not all the metabolites recovered to its original level. This illustrated that high altitude hypoxia has a long-term effect on metabolism. We delineate metabolic profile of plasma under hypoxic exposure, as well as after return to normoxia in physiological states. The differentiated metabolites can be treated as early warning markers of acute injury under high altitude hypoxia exposure at high altitude, which provide new insights into the establishment of health surveillance programmes.

## Supporting information

S1 File (DOCX)
