# Supplementary material for: Metabolomic analysis of human plasma sample after exposed to high altitude and return to sea level
Source: PLoS One. 2023 Mar 29;18(3):e0282301. doi: 10.1371/journal.pone.0282301 (PMC10058093; doi:10.1371/journal.pone.0282301)
Supplement: S1 File — (DOCX) [file pone.0282301.s001.docx]

**Metabolomic analysis of human plasma sample after exposed to high altitude and return to sea level**

Jiayue Gao^1, ¶^, Ming Zhao^1, ¶^, Xiang Cheng^1^, Xiangpei Yue^1^, Fangbin Hao^2^, Hui Wang^2^, Lian Duan ^2^, Cong Han ^2, *^, Lingling Zhu^1, 3, *^

^1^ Beijing Institute of Basic Medical Sciences, Beijing 100850, China

^2^ The Fifth Medical Centre, Chinese PLA General Hospital, Beijing 100071, China;

^3^ Anhui Medical University, Hefei 230000, China

*** Corresponding Authors**

Cong Han, M.D, Ph.D.

Tel: 8610-66947388
Email: hc82225@126.com

Address: No.8 Dong-Da Street, Fengtai District Beijing 100071, China.

Lingling Zhu, M.D, Ph.D.

Tel: 8610-66931315
Email: linglingzhuamms@126.com

Address: No.27, Taiping Road, Haidian District, Beijing, 100850, China.

^¶^These authors contributed equally to this work.


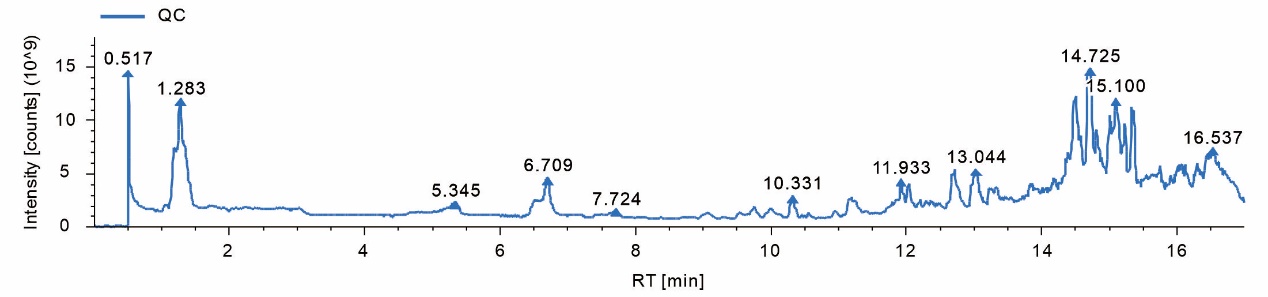


**Figure S1.** Total ions chromatograms (TIC) of plasma sample.

**Table S1** Information of 8 candidate metabolites in QC using LC-MS analysis

| Name | QC1 | QC2 | QC3 | QC4 | QC5 | QC6 | QC7 | QC8 | QC9 | QC10 | QC11 | RSD |
| --- | --- | --- | --- | --- | --- | --- | --- | --- | --- | --- | --- | --- |
| Adenosine | 6.05E+07 | 6.39E+07 | 6.02E+07 | 7.33E+07 | 5.85E+07 | 6.51E+07 | 6.41E+07 | 6.48E+07 | 6.54E+07 | 6.96E+07 | 6.63E+07 | 6.52% |
| Guanosine | 7.30E+06 | 7.85E+06 | 8.74E+06 | 7.97E+06 | 7.90E+06 | 7.93E+06 | 8.12E+06 | 8.56E+06 | 8.06E+06 | 9.00E+06 | 8.38E+06 | 5.82% |
| Inosine | 2.39E+07 | 2.45E+07 | 2.34E+07 | 2.82E+07 | 2.43E+07 | 2.50E+07 | 2.82E+07 | 2.55E+07 | 2.65E+07 | 2.84E+07 | 2.63E+07 | 6.97% |
| Xanthurenic acid | 5.38E+06 | 5.65E+06 | 5.94E+06 | 7.01E+06 | 6.37E+06 | 5.89E+06 | 6.14E+06 | 6.49E+06 | 5.98E+06 | 6.22E+06 | 6.29E+06 | 7.13% |
| 5-OxoETE | 2.02E+08 | 1.16E+08 | 1.60E+08 | 1.48E+08 | 2.44E+08 | 2.31E+08 | 1.47E+08 | 1.31E+08 | 2.60E+08 | 2.64E+08 | 2.02E+08 | 27.98% |
| Indole-3-acetic acid | 1.30E+09 | 1.21E+09 | 1.13E+09 | 1.20E+09 | 1.10E+09 | 1.11E+09 | 1.14E+09 | 1.17E+09 | 1.14E+09 | 1.18E+09 | 1.20E+09 | 4.94% |
| Raffinose | 2.31E+06 | 3.55E+06 | 3.91E+06 | 4.21E+06 | 2.34E+06 | 4.65E+06 | 3.54E+06 | 2.88E+06 | 2.98E+06 | 4.57E+06 | 3.63E+06 | 23.11% |
| Biotin | 1.30E+06 | 1.37E+06 | 1.49E+06 | 1.57E+06 | 1.81E+06 | 1.59E+06 | 1.76E+06 | 1.72E+06 | 1.60E+06 | 1.75E+06 | 2.11E+06 | 5.51% |
